# Toll signals regulate dorsal–ventral patterning and anterior–posterior placement of the embryo in the hemipteran *Rhodnius prolixus*

**DOI:** 10.1186/2041-9139-5-38

**Published:** 2014-10-27

**Authors:** Mateus Berni, Marcio Ribeiro Fontenele, Vitoria Tobias-Santos, Aline Caceres-Rodrigues, Flavia Borges Mury, Raquel Vionette-do-Amaral, Hatisaburo Masuda, Marcos Sorgine, Rodrigo Nunes da Fonseca, Helena Araujo

**Affiliations:** Instituto de Ciências Biomédicas (ICB), Universidade Federal doRio de Janeiro, Av. Carlos Chagas Filho 373, Ilha do Fundão., Rio de Janeiro, 21941-902 RJ Brazil; Institute of Molecular Entomology, INCT-INEM, National Institutes in Science and Technology, Macaé, Brazil; Post-graduate Program in Morphological Sciences, Federal University of Rio de Janeiro (PCM/UFRJ), Rio de Janeiro, RJ Brazil; Laboratório Integrado de Bioquímica Hatisaburo Masuda, Núcleo de Pesquisas Ecológicas de Macaé (NUPEM), Universidade Federal do Rio de Janeiro (UFRJ), Campus Macaé, Av. São José do Barreto, 764, 27937-450 Macaé, RJ, Brazil; Institute of Medical Biochemistry, Federal University of Rio de Janeiro, Rio de Janeiro, RJ, Brazil

**Keywords:** Toll, Dorso-ventral axis, Embryogenesis, NFκB, Hemipteran

## Abstract

**Background:**

Insect embryonic dorso-ventral patterning depends greatly on two pathways: the Toll pathway and the Bone Morphogenetic Protein pathway. While the relative contribution of each pathway has been investigated in holometabolous insects, their role has not been explored in insects with a hemimetabolous type of development. The hemimetabolous insect *Rhodnius prolixus*, an important vector of Chagas disease in the Americas, develops from an intermediate germ band and displays complex movements during katatrepsis that are not observed in other orders. However, little is known about the molecular events that regulate its embryogenesis. Here we investigate the expression and function of genes potentially involved in the initial patterning events that establish the embryonic dorso-ventral axis in this hemipteran.

**Results:**

We establish a staging system for early embryogenesis that allows us to correlate embryo morphology with gene expression profiles. Using this system, we investigate the role of Toll pathway genes during embryogenesis. Detailed analyses of gene expression throughout development, coupled with functional analyses using parental RNA interference, revealed that maternal Toll is required to establish germ layers along the dorso-ventral axis and for embryo placement along the anterior-posterior axis. Interestingly, knockdown of the Toll pathway effector *Rp-dorsal* appears to regulate the expression of the Bone Morphogenetic Protein antagonist *Rp-short-gastrulation*.

**Conclusions:**

Our results indicate that Toll signals are the initiating event in dorso-ventral patterning during *Rhodnius* embryogenesis, and this is the first report of a conserved role for Toll in a hemipteran. Furthermore, as *Rp-dorsal* RNA interference generates anteriorly misplaced embryos, our results indicate a novel role for Toll signals in establishment of the anterior-posterior axis in *Rhodnius*.

**Electronic supplementary material:**

The online version of this article (doi:10.1186/2041-9139-5-38) contains supplementary material, which is available to authorized users.

## Background

Morphological diversity in the class Insecta is reflected in its different developmental modes. In addition to attaining the adult form directly (ametabolous) through complete (holometabola) or incomplete (hemimetabola) metamorphosis, the size of the embryonic rudiment relative to the egg determines many of the morphogenetic events that ensue. Insect eggs are divided into long, intermediate, and short germ types according to the size of the embryonic rudiment and the number of segments that are specified before gastrulation (reviewed in [1]). This diversity of developmental modes poses challenges for understanding the molecular basis of insect development.

As a first step in embryonic development, the dorso-ventral (DV) and anterior-posterior (AP) axes are specified, leading to patterned embryonic gene expression. In insects, patterning along the DV axis is required to subdivide mesodermal and ectodermal embryonic tissues and to drive formation of extraembryonic membranes. Two major pathways have been described that pattern the DV axis in arthropods: the Toll receptor pathway and the Bone Morphogenetic Protein (BMP) pathway. Their respective contribution to DV patterning, as well as their maternal versus zygotic functions, varies among species (see references in [2]). For instance, in the long-germ diptera *Drosophila melanogaster*, the maternal Toll pathway establishes domains of zygotic gene expression along the DV axis. *Drosophila* Toll regulates the activity of the NFκB family downstream effector protein Dorsal (Dl). Upon activation of the Toll pathway, Dl is no longer targeted for degradation by the Cactus/IκB inhibitor and can translocate to the cell nuclei to regulate the expression of target genes, including localized expression of the BMP pathway genes *decapentaplegic* (*dpp*) and *short gastrulation* (*sog*) (see references in [3]). In contrast, in the beetle *Tribolium castaneum*, which presents a more ancestral short-germ mode of embryogenesis, elements of the Toll pathway are maternally provided but most DV patterning occurs at the zygotic level [4–6]. Furthermore, BMPs play a key role in setting up the DV axis in *Tribolium*, and *Tc-Toll* does not regulate *Tc-dpp* expression [5, 7].

Based on the conserved role of BMPs in patterning the bilaterian DV axis, it has been hypothesized that Toll was co-opted from a more ancestral immune function for DV patterning in insects [2]. Arthropod DV patterning has been studied in chelicerates such as the spider model *Parasteatoda tepidariorium*[8] and holometabolous insects such as *Nasonia vitripennis*, *T. castaneum*, and *D. melanogaster* (reviewed in [2, 9]). Recently, the role of Toll and BMP pathways has been analyzed in two long germ hymenopteran species, the bee *Apis mellifera* and the wasp *N. vitripennis*[10, 11]. In *N. vitripennis* the BMP gradient and its dorsally expressed target genes are not regulated by Toll signaling [11]. In *A. mellifera* Toll and BMP are involved not only in DV but also in AP axis establishment [10]. However, embryonic DV patterning has not been investigated in hemimetabolous insects. Considering the lack of knowledge about DV patterning in insects with a more direct (hemimetabolous) type of development, we set out to investigate the relative contributions of the Toll and BMP pathways to DV patterning in the hemimetabolous insect *Rhodnius prolixus*, which develops with an intermediate germ type of embryogenesis.

The hemiptera *Rhodnius prolixus* is an important vector of Chagas disease, which affects over 8 million people in Latin America [12]. As an established model for insect physiology, recent genomic analyses are contributing to a global understanding of *R. prolixus* genome size and organization, and tissue-specific sequences are being identified [13–15]. This community effort also provides the molecular basis for investigating the function and network of interactions between developmental genes.

*Rhodnius* egg production depends on blood feeding. After each blood meal, a female lays up to 40 eggs [16, 17]. Three extracellular membranes plus a hard chorion protect the resulting embryos from desiccation [18]. While several morphological features of *Rhodnius* embryonic development have been described [18–21], the molecular aspects that regulate *Rhodnius* development remain unexplored. This is especially true for the initial embryonic stages where axial patterning events must take place, and before overall embryo morphology is set. The long-lasting effects of parental double-stranded RNA interference (pRNAi) assays recently described for this species now provide a tool for addressing these questions [22–27].

In this study, we established a staging system for early *Rhodnius* embryogenesis, which enabled us to correlate embryo morphology with gene expression, and provided a framework for functional studies. Next, we investigated whether the genes associated with the Toll and BMP pathways are expressed in the *Rhodnius* embryo, which revealed that *Rp-Toll* and *Rp-dl* are maternally and zygotically expressed. With this information, we performed functional analyses for Toll pathway elements. Our results suggest that the Toll pathway acts to specify germ layers along the DV axis and to localize the embryo along the long axis of the *Rhodnius* egg. Thus, our results implicate Toll signaling as a central pathway in *Rhodnius* embryonic development.

## Methods

### Insect rearing, tissue fixation, and histological sections

*Rhodnius* rearing was performed as previously described [26, 27]. Ovary collection and dissection were performed using standard protocols available for *D. melanogaster*. Briefly, ovary fixation was performed at room temperature with 4% paraformaldehyde (PFA) for 1 hour shortly before Toll immunostaining (see below). For embryonic fixation we have established a new protocol involving trypsin digestion, sonication, and increased fixation time (detailed protocol available upon request). Briefly, the embryos were fixed at 4% PFA in phosphate buffer pH 7.4 with Tween-20 0.1% at room temperature for 24 hours, sonicated for 2 minutes, and then treated with trypsin for chorion permeabilization for 5 minutes. The next step was post-fixation for 12 hours with 4% PFA. After manual dechorionation with fine forceps, embryos were stained with DAPI (1 μg/μL). For frozen histological sections, embryos were fixed overnight in 4% PFA, washed in PBS and included in OCT (Tissue-Tek, Sakura Finetek, Torrance, CA, USA) in 2-hour steps, from 25% to 100% OCT. Sections (20 μm) were obtained in a CM 1510S Leica cryostat (Leica, Wtzler, Germany).

For ovary immunostaining, egg chambers were fixed for 20 minutes in 4% PFA, washed in phosphate buffer pH 7.4 with Tween-20 0.1%, blocked with 0.1% normal goat serum and incubated overnight at 4°C with primary antibody Toll d-300 (Santa Cruz Biotechnology, Santa Cruz, CA, USA, 1:50 dilution). Detection was performed with secondary anti-rabbit 488 (1:1000, 1 hour/room temperature; Alexa Fluor, Invitrogen, Life Technologies) and counterstained with Alexa 546 conjugated phalloidin (1:100, 1 hour/room temperature; Invitrogen) and DAPI. Images were obtained with a Leica SP5 confocal microscope.

### Identification of Bone Morphogenetic Protein- and Toll-related genes in *R. prolixus*genome and phylogenetic analysis

*D. melanogaster* or *T. castaneum* protein sequences were used as BLAST queries in the *Rhodnius prolixus* unpublished genome (http://genome.wustl.edu/genomes/detail/rhodnius-prolixus/). BLASTs were also performed in the recently published *R. prolixus* gut transcriptome [14]. After manual curation, protein sequences were aligned using the CLUSTALW algorithm available in the MEGA6 package [28]. The best substitution model for this set of Toll protein sequences (LG + G) was calculated also in MEGA6. Phylogenetic analysis of Toll-related genes was performed using a maximum likelihood method with 100 replicates for bootstrap, complete deletion of gaps and using the nearest-neighbor-interchange method.

Accession numbers for the genes analyzed were: EF1: RPRC015041; 18S, GenBank ID: AJ421962.1; *Rp-dpp*, GenBank ID: GU906792.1; *Rp-dl*, GenBank ID: ABU96698.1; *Rp-sog*: RPRC015365-RA *Rp-cact*: Asb-31044 (VectorBase). Accession numbers for Toll genes are in main text, RPRC accession numbers are from Vectorbase (https://www.vectorbase.org/).

### Quantitative PCR

Total RNA was isolated using TRIzol reagent (Invitrogen) from previtellogenic, vitellogenic, or choriogenic ovaries, and embryos collected for 6-hour time windows and aged to the periods described in the text. RNA was treated with RNAse free TURBO™ DNAse (Ambion, Life Technologies), and cDNA was synthesized from 1 μg total RNA using the Multiscribe kit (Applied Biosystems.Real-time quantitative PCR was performed on a StepOnePlus real-time PCR system using power SYBR-green PCR master mix (Applied Biosystems, Foster City, USA). The comparative Ct method [29, 30] was used to compare changes in gene expression levels. The ribosomal *18S* (*18S*) and *Elongation factor 1* (*Ef1*) genes were used as endogenous reference genes ( [31] and Additional file 1). Primer pairs are listed in Additional file 1. All primers were designed with Primer 3 (http://frodo.wi.mit.edu/primer3/) and checked for dimer formation with Oligoanalyzer software (http://www.idtdna.com/analyzer/applications/oligoanalyzer/)*.* All quantitative real-time PCR experiments were performed in triplicate, with four technical replicates. Bars in graphs correspond to standard error.

### Parental RNA interference

Double-stranded RNA was synthesized from PCR products containing T7 promoter sequences at both ends as previously described [9]. Briefly, two successive PCRs were performed; the first to amplify *Rp-dl* and *Rp-cact* from the cDNA and the second PCR added T7 promoter sequences at both ends. Primer pairs are listed in Additional file 1. BLASTn against the *Rhodnius prolixus* genome did not detect unrelated sequences similar to the selected regions. The unrelated *MalE* gene (Gene ID: 948538), present in *Escherichia coli,* but absent from the *R. prolixus* genome, was used as a dsRNA control (*dsMalE*) for the off-target effects of dsRNA. The *MalE* 800 bp fragment was amplified from the Litmus 281-plasmid (New England Biolabs, Iswich, MA, USA) with a single primer (T7, 5-TAATACGACTCACTATAGGG-3) specific for the T7 promoter sequence that is on both sides of the *MalE* sequence. The different dsRNA concentrations used during the studies are described in Results. dsRNA injections into *R. prolixus* female virgins followed recently published protocols [23]. Two days after dsRNA injection the females were fed with rabbit blood. Oviposition and egg collection were initiated 1 week after blood feeding. Eggs were collected, counted, and the hatch rate defined after 20 days at 28°C, to make sure all surviving embryos had hatched.

### Western blot

Proteins were separated under SDS-PAGE and analyzed by Western blots using standard protocols. PVDF membranes were blocked with 5% bovine serum albumin and incubated overnight at 4°C with primary antibody Toll d-300 (Santa Cruz Biotechnology, 1:50 dilution). Proteins were detected with peroxidase conjugated secondary antibody (Vector; 1:4000) and Pierce ECL Western Blot Substrate (Life Technologies).

## Results

### Establishing a staging system to study *Rhodnius prolixus*early embryonic development

**Figure 1:**
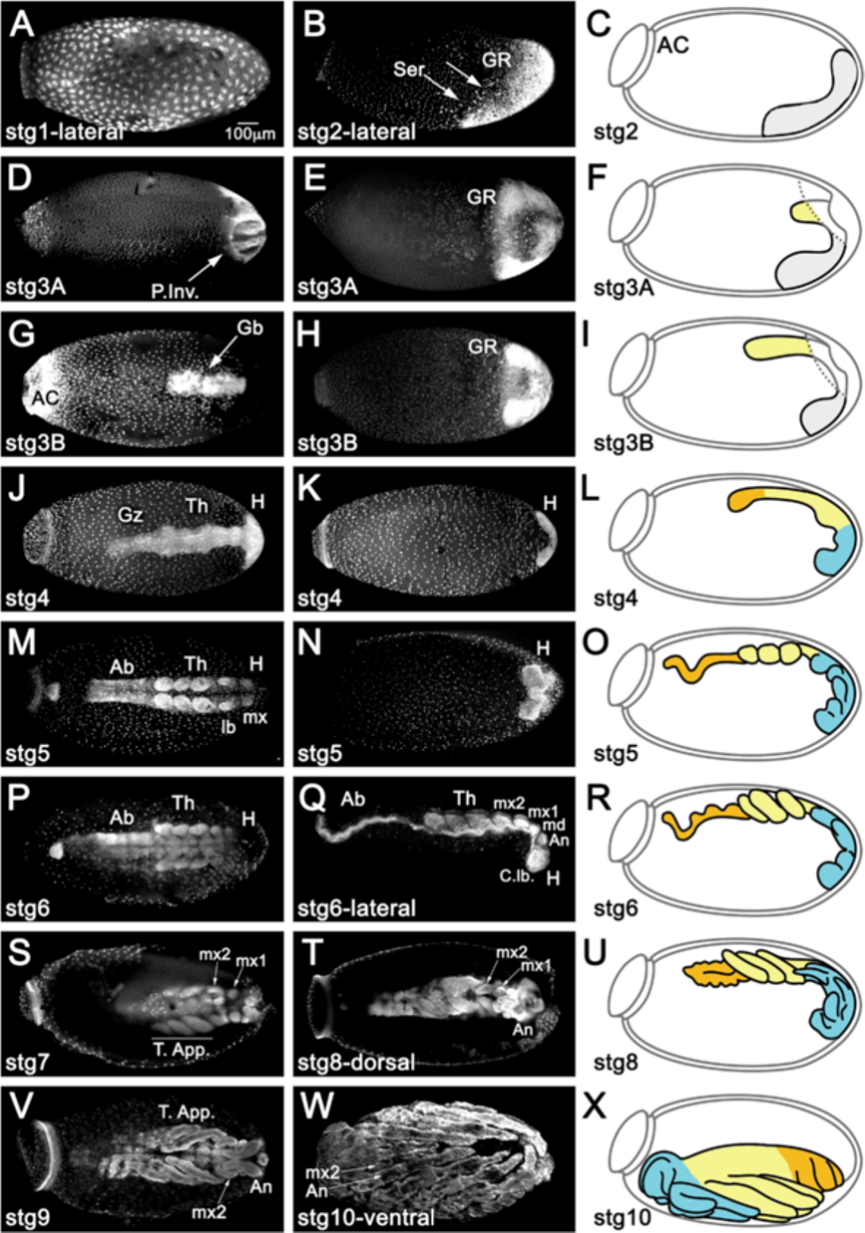
**Prospective patterning stages in** ***Rhodnius*** **early embryogenesis.** Nuclear staining for fixed embryos at the designated stages. Left and mid panel images are taken from the dorsal and ventral egg surface, respectively, unless stated otherwise. Drawings correspond to graphical representations of the stage in lateral views. **(A)** Cleavage stage embryo with nuclei at the periphery. **(B,C)** As the blastoderm develops, nuclei concentrate posteriorly (arrows) to form the germ rudiment (GR, grey in C). Serosal cells (Ser) are placed dorso-anteriorly. **(D-F)** During gastrulation, posterior invagination (P.Inv) of the GR takes places dorsally and **(G-I)** the germ band (Gb) extends anteriorly, while on the ventral surface the non-invaginated surface GR (grey in F) forms a horseshoe. Dashed lines in **(F)** and **(I)** correspond to the tissue under which the Gb invaginates. **(J-L)** Once gastrulation is complete, the head (H) and thorax (Th) regions are visible, as well as a growth zone (Gz) from which abdominal structures develop. **(M-O)** The embryo has grown to its full length. H and Th are segmented, but not the abdominal region (Ab). **(P-R)** Segmentation is complete. Ab is segmented. The abdominal caudal flexure is immersed in the yolk in (M-R) and consequently not visualized in superior optical sections. **(S,T,V)** Growth stages in dorsal views. Cuticle fluorescence adds to and takes over nuclear staining with time. **(S)** Thoracic appendages (T. app.) larger than head appendages. Maxilar appendages mx1 and mx2 are distinguished. **(T,U)** T. app. curved over ventral surface of the embryo. Mx1 and antennal appendages (An) grow. **(V)** Mx1 are hidden by An. Throughout the above stages the DV and AP axes of the embryo are inverted respective to the egg. **(W,X)** During katatrepsis the embryo moves backwards. As a result the embryo and egg axes coincide. Throughout stage 10 the embryo also undergoes dorsal closure. Egg anterior cap (AC); md: mandibule; C. lb.: cephalic lobes. Magnification Bar in **(A)** is valid for all panels.

The hemiptera *R. prolixus* develops from an intermediate germ band, where the anlagen of the head and thoracic segments are established before gastrulation, while the abdominal segments are generated by a secondary process from a posterior growth zone. After 29 days at 21°C or 14 days at 28°C of embryonic development, a nymph hatches as a miniaturized version of the adult ( [20], this report). Although embryogenesis in this species has been previously characterized [19–21, 32] we found it necessary to establish a staging system for the early embryonic stages in order to define windows for morphological and molecular analysis. Thus, we developed a fixation protocol that allows us to highlight the morphogenetic events that accompany embryogenesis from syncytial blastoderm until late stages where the cuticle is secreted (Figure 1). We divided embryogenesis into five main periods based on developmental hallmarks that have been shown to require specific gene networks in other insects. These periods included: cleavage, blastoderm, gastrulation and germ band extension, segmentation, and growth. Stages were defined as 12-hour windows, and further split into 6-hour windows for the initial stages (1 to 3) until the completion of gastrulation.

DV polarity of the *Rhodnius* egg is easily identified immediately after oviposition ( [19, 20], this report) due to the oblique position of the anterior cap (Figure 1C). During Cleavage, the *Rhodnius* embryo develops as a syncytium (from 0 to 12 hours), with nuclear cleavages initially taking place at the center of the egg (0 to 6 hours, stage 1A). After migrating to the periphery (6 to 12 hours, stage 1B), two cell populations begin to segregate: the small syncytial blastoderm nuclei and the putative serosal cells [33, 34]. The syncytial nuclei concentrate at the posterior pole, forming the germ rudiment (GR), which corresponds to the embryonic rudiment plus amniotic rudiment [33, 35] (stage 2).

During the Blastoderm period (12 to 24 hours) membranes form around the blastoderm nuclei (by 18 hours, stage 2B; Figure 1B,C) and, shortly after, the not yet invaginated ventral GR is visible as a U-shaped cubical epithelium curved around the posterior pole, likely corresponding to the prospective head lobes. Germ cells appear at this stage, projecting into the yolk [20, 32].

At 24 hours (stage 3A; Figure 1D-F) gastrulation takes place, which is characterized by the invagination and inward proliferation of GR cells on the dorsal side and by the movement of the ventral part of the GR towards the posterior pole (characterizing immersion anatrepsis [33]). As a result of this movement, the amnion is formed and the embryo proper extends with the anterior cephalic end at the posterior region of the egg (stage 3B; Figure 1G-I). Thus, by the end of gastrulation the embryo and egg AP axes are inverted relative to each other [20, 32]. In addition, the germ band is inverted relative to the egg DV axis and placed on the dorsal side of the egg, which differs from the pattern displayed by another hemiptera species, *Oncopeltus fasciatus*[36, 37].Following germ band extension, morphological Segmentation begins in the anterior region of the embryo (36 hours, stage 4; Figure 1J-L). At 48 hours (stage 5; Figure 1M-O), cephalic and thoracic appendages are visible. Segmentation of abdominal regions is visible at 60 hours (stage 6; Figure 1P-R).

Subsequent stages characterize the Growth phase, where the different appendages develop and most of the embryo, especially posterior regions, is immersed in yolk (stages 7 to 9, 72 to 108 hours; Figure 1S-V). During stage 10 the embryo undergoes the relatively rapid process of Katatrepsis (Figure 1W,X), where the embryo emerges from the yolk by rotating backwards 180° [32]. As a result, the cephalic region of the embryo now aligns itself with the anterior of the egg. Similar movements are observed in most hemimetabolous insects and require the presence of the serosal membrane [33], which in *Rhodnius* contracts at the anterior side of the egg [19, 32]. After stage 7, secretion of the cuticle [26, 32] disturbs tissue visualization with fluorescent nuclear staining.

In conclusion, using our fixation and staging protocol with whole mount nuclear stains, we were able to easily assess the general morphology of the embryo throughout all stages and its position relative to the axis of the egg, as well as the processes of germ band extension and segmentation (Table 1). The large amount of yolk relative to blastoderm nuclei, however, made it difficult for us to perform a detailed morphological analysis during the pre-blastoderm stage.

**Table 1:** **Major features of** ***Rhodnius*** **embryonic development until katatrepsis**

Period	Stage	Developmental time at 28°C		Characteristic features in whole mounts
		Hours	%*	
Cleavage	1A	0–6	1.8	Nuclei divide internally
	1B	6–12	3.6	Nuclei reach the periphery
Blastoderm	2A	12–18	5.3	Formation of germ rudiment
	2B	18–24	7.1	Nuclei progressively concentrate at egg posterior
Gastrulation	3A	24–30	8.9	Germ rudiment invagination begins
	3B	30–36	10.7	Germ band extension
Segmentation	4	36–48	14.3	Anterior segmentation (in head and thorax) begins
	5	48–60	17.9	Appendage buds appear; non-segmented abdomen
	6	60–72	21.4	Appendages grow;
				segmented abdomen; neural groove evident
Growth	7	72–84	25	Thoracic appendages elongate, bigger than head appendages
	8	84–96	26.6	Antennal appendages grow over first maxillary appendages
	9	96–108	32	First maxillary appendages hidden by antennal appendages; embryo shortens and widens (germ band retraction)
Katatrepsis and Dorsal Closure	10	108–132	35	At the end of katatrepsis, embryo and egg anterior-posterior axes coincide; embryo closes dorsally

*% refers to total embryonic development at 28°C.

**Figure 2:**
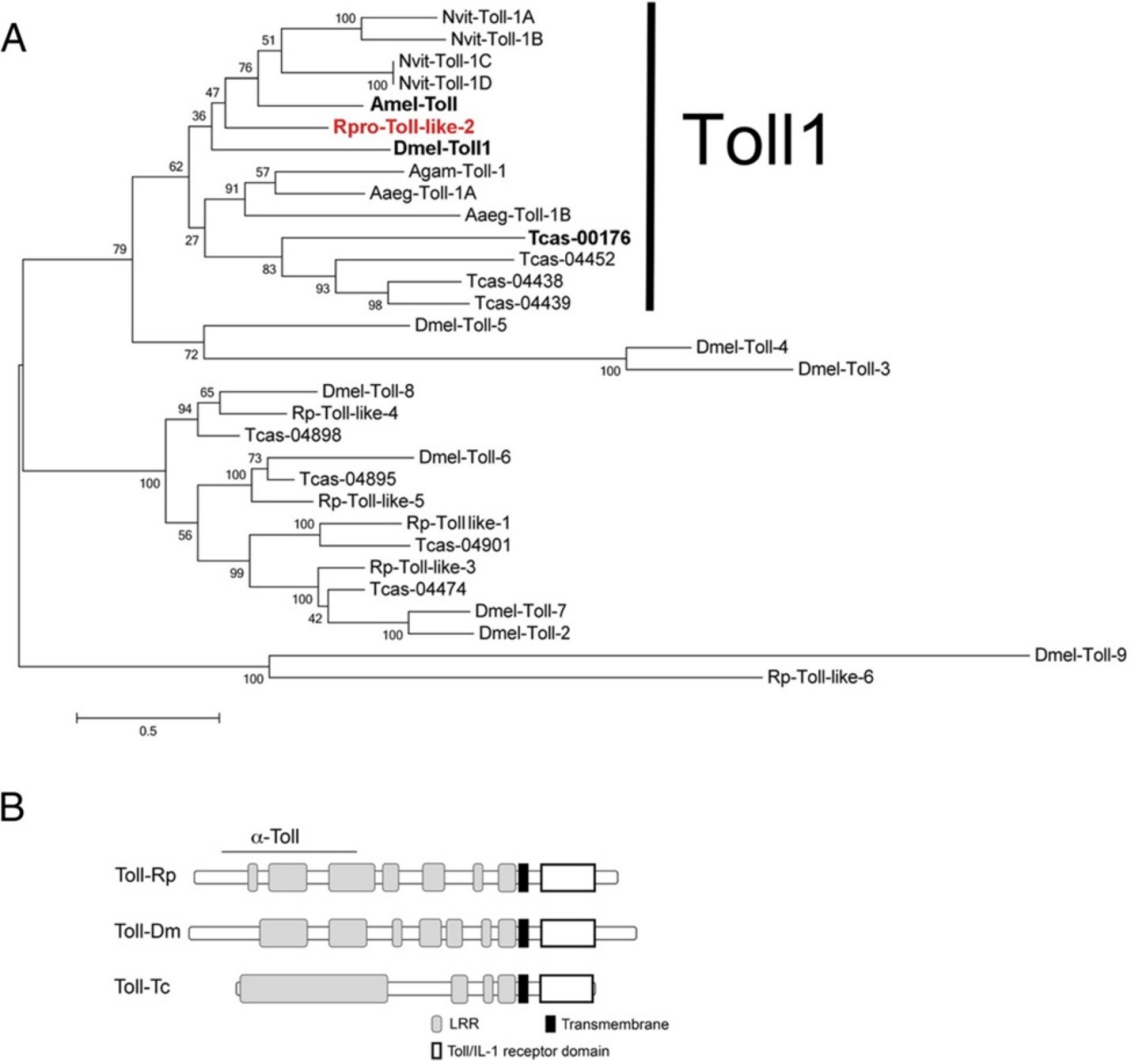
**Rp-Toll-like-2 is related to other Toll involved in dorso-ventral patterning. (A)** Phylogenetic tree illustrating the relationship between *Rp* Tolls and other Toll-like receptors. **(B)** Predicted protein structure for Rp-Toll-like-2 (Rp-Toll), compared to Dm-Toll and Tc-Toll, with region recognized by anti-Toll d300 (α-Toll). Sequences used for phylogenetic tree reconstruction were: *Nvit-Toll-1A*: XP_001604577.1*; Nvit-Toll-1B*: XP_001604871.2; *Nvit-Toll-1C*: XP_001604880.2; *Nvit-Toll-1D:* XP_003425965.1; *Dmel-Toll*: CG5490-PC, *Dmel-Toll2*: AAF57509.1, *Dmel-Toll-3*: AAF86229.1, *Dmel-Toll-4*: CG18241, *Dmel-Toll-5*: AAF86227.1, *Dmel-Toll-6*: CG7250, *Dmel-Toll-7*: CG8595-PA, *Dmel-Toll-8*: AAF862241, *Dmel-Toll-9*: CG5528, *Tcas-04901*: XP_972312.1, *Tc-00176*: XP_967154.1, *Tcas-04438*: XP_967796.1, *Tcas-04439*: XP_967716.2, *Tcas-04452*: XP_973926.2, *Tcas-04474 XM_967316.2 Tcas-04895*: XP_971999.1, *Tcas-04898:* XP_008200897.1, *Tcas-04901:* XP_972312.1|, *Rpro-Toll-like-1* [VB: RPRC07390], *Rpro-Toll like-2* [VB: RPRC 009262], *Rpro-Toll-like-3* [VB: RPRC015296], *Rpro-Toll like-4* [VB: RPRC004104], *Rpro-Toll-like-5:* GL562958 (a contig, not a predicted transcript), *Rpro-Toll-like-6* [VB: RPRC001608-RA], *Agam-Toll1*: AAL37901.1, *Aaeg-Toll1A*: AAEL000057, *Aaeg-Toll1B: XP_001658507.1*, *Amel-Toll: XP_006562783.1* (*Nvit*: *Nasonia vitripennis*; *Tcas*: *Tribolium castaneum*; *Dmel*: *Drosophila melanogaster; Rpro*: *Rhodnius prolixus; Agam*: *Anopheles gambia; Isca*: *Ixodes scaputari; Aaeg*: *Aedes aegypti*). Genes functionally analyzed for a role in dorso-ventral patterning are displayed in bold, Rp-Toll-like-2 (Rp-Toll) functionally analyzed in this study is in red.

### Several Toll and Bone Morphogenetic Protein pathway components are conserved in *Rhodnius*

In order to address which genes are required to pattern the *Rhodnius* DV axis, we first sought to identify Toll and BMP pathway genes that may be expressed during embryogenesis. We recently identified complete or partial sequences for genes in both pathways, including six different Toll sequences in the *R. prolixus* genome. Expression was confirmed for most of these in the recently published *R. prolixus* transcriptome raw data [14]. Phylogenetic analysis suggests that *Rp-Toll-like-*2 (RPRC009262), herein referred to as *Rp-Toll*, is a *Drosophila* Toll ortholog (Figure 2A). The *Rp-Toll* sequence was aligned to *Drosophila* and *Tribolium* Toll proteins, which revealed that this sequence comprises a transmembrane region, part of the extracellular segment and most of the intracellular domain that interacts with downstream elements (Figure 2B and Additional file 2). The predicted *Rp-Toll* protein sequence presents 44% sequence identity with *Drosophila* Toll, and most of this identity covers the region identified by the anti-Dm-Toll antiserum used in this study (Figure 2B and Additional file 2).

Sequences coding for a Dorsal transcription factor (an NFκB/c-Rel protein activated by Toll) have been previously identified in *R. prolixus*[38]. These authors detected *Rp-dl* transcripts in the embryo (*dl,* 1A) as well as adult salivary glands, midgut, and fat body (*dl,* 1B and 1C). Accordingly, we identified an inhibitory *cactus*/*IkB* ortholog that is expressed in *R. prolixus* ovaries, embryos, fat body, and in the digestive tract.

BMP family proteins signal through heterodimeric transmembrane receptors to induce phosphorylation of R-Smads, leading to activation of Co-Smads and regulation of gene transcription. Classically, BMPs are regulated in the extracellular space by binding to Chordin (Chd)/Short gastrulation (Sog) antagonists. We have identified in the *R. prolixus* genome two sequences coding for BMP ligands (*Rp-dpp* and *Rp-gbb*) and one sequence for the antagonist Sog (*Rp-sog*). Furthermore, one type I (*Rp-tkv*) and one type II (*Rp-punt*) BMP receptor, two R-Smads (*Rp-mad* and *Rp-mad-like*), and one Co-Smad (*Rp-medea*) are represented as expressed sequences, implying that all elements necessary for BMP signaling are present (Mesquita and colleagues, manuscript in preparation). Given that Dpp/BMP and Sog/Chd sequences have been previously associated to embryonic DV patterning in arthropods and vertebrates, we chose to investigate these homologs in greater detail.

### Expression of Toll and Dpp pathway genes varies throughout embryonic development

**Figure 3:**
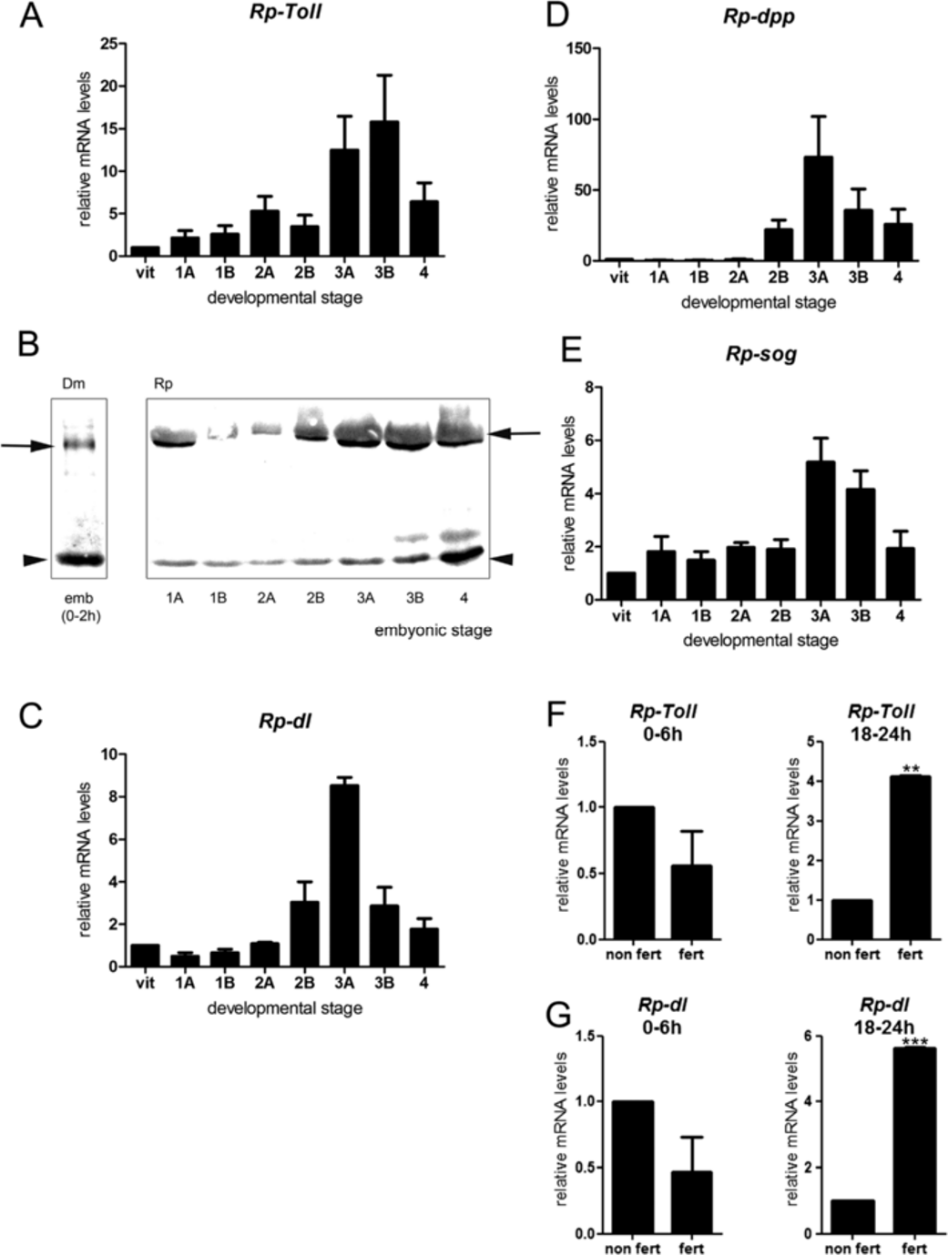
**Toll and Bone Morphogenetic Protein pathway genes are dynamically expressed throughout embryonic development.** Normalized mRNA levels for **(A,F)** *Rp-Toll*, **(C,G)** *Rp-dl*, **(D)** *Rp-dpp*, and **(E)** *Rp-sog* in vitellogenic ovaries (vitelo), embryonic stages (stages 1 to 4, 0 to 48 hours), or unfertilized eggs **(F,G)**. Differences in gene expression throughout embryonic development were significant for all genes between preblastoderm (0 to 12 hours) and gastrulation stages (18 to 30 hours) (one-way analysis of variance, *p* <0.05). **(B)** Toll protein levels vary throughout *Rhodnius* development. **(F,G)** Differences in mRNA levels for **(F)** *Rp-Toll* and **(G)** *Rp-dl* in fertilized (fert) versus unfertilized (non fert) 0- to 6-hour or 18- to 24-hour eggs were significant: **p <0.01, ***p <0.001 by paired *t*-test. Use of appropriate reference genes was defined as in Additional file 1. Dm, *D. melanogaster*; Rp, *R. prolixus*.

In order to investigate whether Toll and BMP pathway genes may play a role in embryogenesis, we used quantitative real-time PCR to characterize their expression from embryonic stages 1 to 4. Given that early embryogenesis frequently depends on maternal messages, we also analyzed expression during oogenesis. For all time points analyzed, we normalized mRNA levels to two reference genes, which revealed similar variation patterns (see Methods for details) [31]. We found that *Rp-Toll* mRNAs are present during the early stages of cleavage and increase during formation of the GR (Figure 3A). *Rp-Toll* is also expressed in the ovaries, indicating that the mother may provide the messages detected during stage 1A (Figure 3A). We observed similar *Rp-Toll* mRNA levels in 0- to 6-hour embryos and unfertilized eggs, confirming that these messages are maternally provided (Figure 3F).

**Figure 4:**
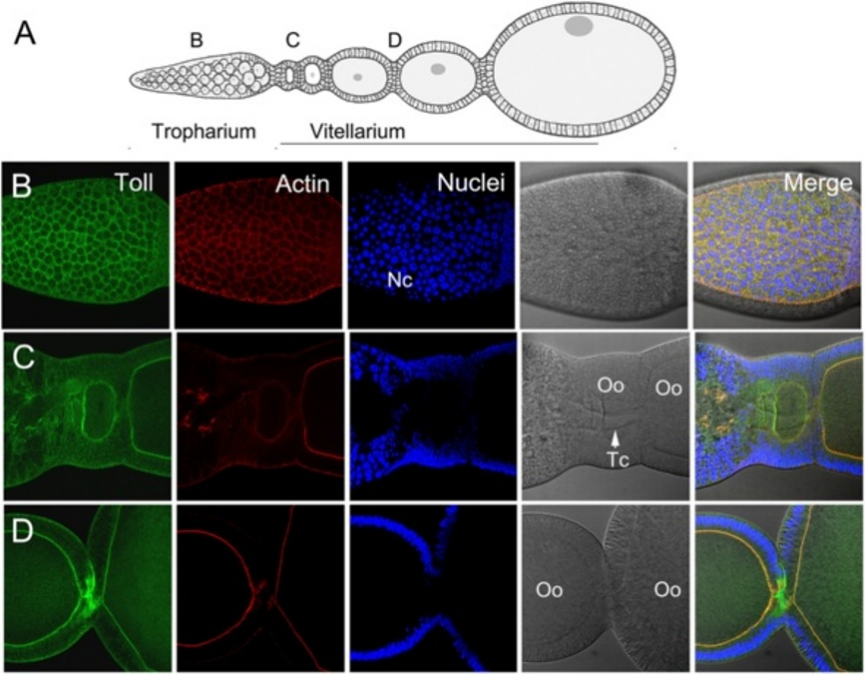
**Toll protein is maternally delivered to developing oocytes. (A)** *Rhodnius* oogenesis showing the tropharium **(B)**, early **(C)**, and late **(D)** oocytes in vitelarium. Chorionic stages are not displayed. **(B)** Toll protein is present in follicle cells of the tropharium. **(C)** Toll in early oocytes is likely delivered from nurse cells through trophic cords. **(D)** Protein in late oocyte cytoplasm and plasma membrane. Toll protein in green, actin to reveal cell perimeter in red, nuclei DAPI stain in blue. Nc: nurse cells; Oo: oocyte; Tc: trophic cord.

We then sought to determine the expression of the Toll protein during *Rhodnius* embryogenesis. To this end, we employed a specific antibody directed against the *Drosophila* Toll (see Methods). In agreement with the high degree of sequence identity between Rp-Toll and Dm-Toll, we show that the Dm-Toll antibody crossreacts with the *Rhodnius* homologous protein in Western blot assays. Toll protein expression during embryogenesis (Figure 3B) is consistent with *Rp-Toll* mRNA expression, with the highest protein levels observed during early cleavage and during gastrulation through germ band extension. However, the low mRNA levels observed during embryonic cleavage suggest that the large amount of protein at this stage derives from maternal *Rp-Toll* messages that are delivered to the embryo where high rate translation takes place. Alternatively, maternal protein is loaded into the oocyte and future embryo. The latter hypothesis is consistent with the ovarian Toll protein pattern observed in this study. To investigate whether Toll protein in early cleavage embryos could result from protein transferred from the mother during oogenesis, we performed immune labeling in *Rhodnius* egg chambers. We observed Rp-Toll protein in the tropharium and vitellarium, in the plasma membrane of follicle cells and developing oocytes (Figure 4). Staining in oocytes probably originated from the nurse cells through trophic cords. This staining is specific as it was lost in *Rp-Toll* knockdowns (Additional file 3).

**Figure 5:**
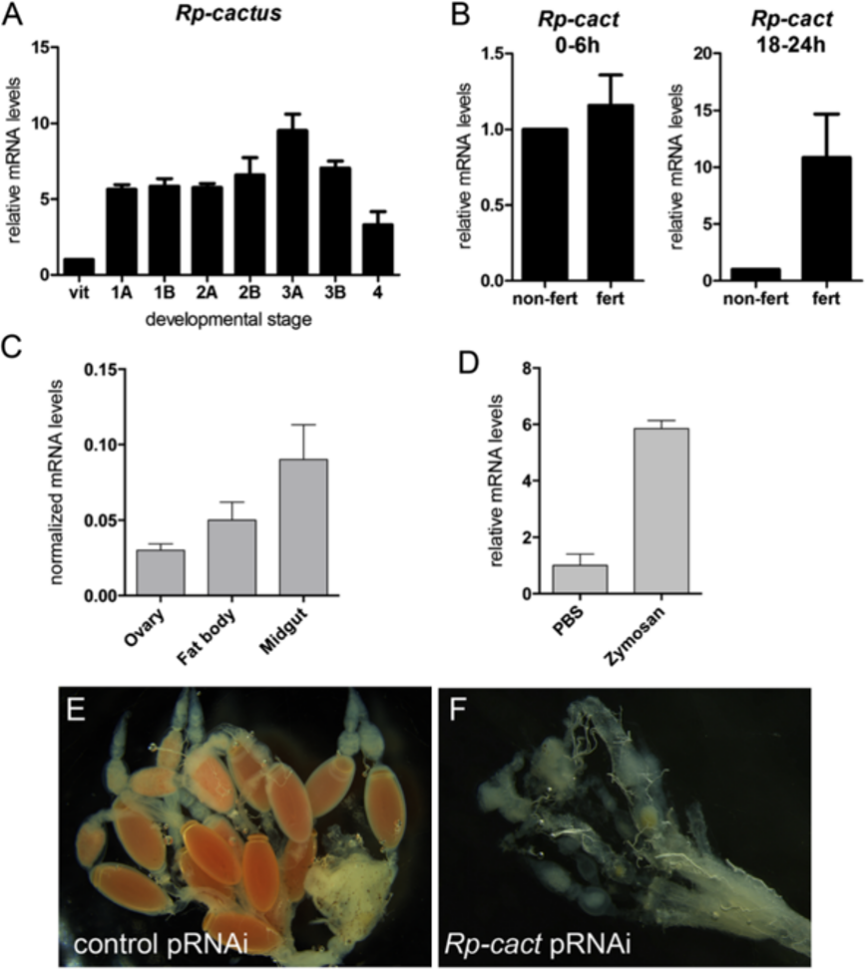
***Rp-cact*** **expression and knockdown phenotypes. (A)** Normalized mRNA levels for *Rp-cact* show small variability throughout embryonic development. **(B)** *Rp-cact* mRNAs in stage 1A embryos (0 to 6 hours) are mostly maternally provided, while gastrulation stage 2B (18 to 24 hours) mRNAs are generated zygotically, since levels of the former are unaltered, while the latter are decreased in unfertilized eggs (non fert). **(C)** *Rp-cact* is also expressed in the immune tissues of the midgut and fat body. **(D)** In immune tissues such as the gut, *Rp-cact* levels respond to activation of the Toll pathway using zymosan. **(E,F)** Ovaries resulting from control *MalE* **(E)** or *Rp-cact* **(F)** pRNAi show that egg chambers do not develop in the *Rp-cact* knockdown.

Since it is a putative effector of the Toll pathway, we investigated the expression of *Rp-dl* during oogenesis and embryogenesis (Figure 3C). Similarly to *Rp-Toll*, *Rp-dl* is expressed during oogenesis and low-abundance *Rp-dl* transcripts are detected during cleavage stages. Significant expression is seen during stages 3A and B of gastrulation, which coincides with the increase in Toll expression (Figure 3A). At 18 to 24 hours (stage 2B), *Rp-Toll* and *Rp-dl* transcript levels are increased relative to unfertilized eggs (Figure 3F-G). Thus, peak mRNA levels at the subsequent stage 3 correspond mostly to zygotic expression. Accordingly, the putative NFκB inhibitor *Rp-cact* is expressed in ovaries and displays constant expression levels throughout embryogenesis Figure 5A. It is also expressed in the fat body and midgut Figure 5C, two important tissues for the innate immune response. These results suggest that *Rp-cact* may respond to maternal and zygotic Toll signals in the embryo.

As part of the BMP pathway*, Rp-dpp* expression, on the other hand, was only observed at the onset of gastrulation (Figure 3D, Additional file 4). This pattern is different from *Rp-sog* (Figure 3E), which is expressed at low levels during early stages and coincides with peak *Rp-Toll* and *Rp-dl* zygotic expression. High *Rp-dpp* and *Rp-sog* mRNA levels in stages 2B and 3 result from zygotic expression, given that these peaks are not observed in unfertilized eggs (data not shown). Therefore, it is unlikely that maternal *Rp-dpp* mRNA provides information for early patterning in *Rhodnius*.

### *Rp-dl*knockdown alters embryonic patterning

**Figure 6:**
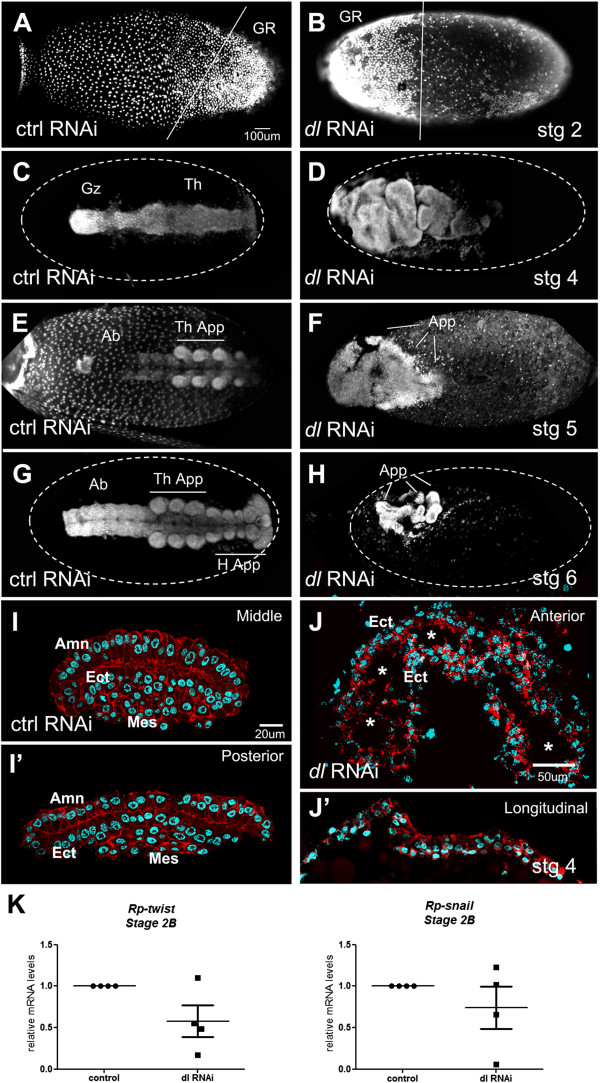
***Rp-dl*** **knockdown embryonic phenotypes.** Embryos resulting from control **(A,C,E,G,I)** or *Rp-dl* **(B,D,F,H,J)** parental RNA interference (pRNAi). **(A,B)** At the blastoderm stage control (ctrl) embryos present a posteriorly localized germ rudiment (GR) that is diagonally displayed (A, as in Figure 2B), while the *Rp-dl* RNAi GR is anteriorly localized and perpendicular to the egg’s long axis. **(C,D)** After gastrulation the head, thorax (Th), and growth zone (Gz) are seen in control **(C)** *MalE* RNAi embryos, but are not distinguished in **(D)** the *Rp-dl* RNAi. **(E,F)** Stage 5 and **(G,H)** stage 6 embryos show the correct formation of appendages in control (E,G) *MalE* RNAi embryos, while **(F,H)** *Rp-dl* RNAi embryos present only appendage-like structures (App.) that are seen in 25% of cases. These structures cannot be identified as head (H.App.) or thoracic appendages (Th. App.). Ab, abdomen. **(I-J’)** After gastrulation, amnion (Amn), ectodermal (Ect) and mesodermal (Mes) layers are distinguished in control embryo transverse sections stained with Alexa 647-phalloidin and nuclear Hoescht **(I,I’)**. Shown are embryo sections in the middle **(I)** and posterior **(I’)** regions of the egg. *Rp-dl* RNAi **(J,J’)** embryos placed at the egg anterior form a hollow tube (Ect) with no distinguishable mesodermal layer, as seen in transverse **(J)** and longitudinal **(J’)** sections. Note that these embryos are localized adjacent to the egg surface. The asterisks in J point to the hollow embryo interior. Lateral view of embryos in (**A,B;** A slightly tilted ventrally), dorsal view in **(C-H)**. However, embryo dorso-ventral placement in *Rp-dl* RNAi is randomized. **(K)** Relative mRNA levels for mesodermal genes in control and *Rp-dl* RNAi embryos (four biological replicates). *Rp-twist* and *Rp-snail* show a tendency to decrease in *Rp-dl* knockdowns.

Based on the pattern of *Rp-Toll* and *Rp-dl* expression in early embryos, we decided to investigate their putative functional role in early embryonic patterning. To this end, we performed pRNAi assays for *Rp-dl*, the sole Toll pathway effector. Females were injected with different concentrations of *Rp-dl* or the unrelated *MalE* dsRNA control (as described in [26, 39]). Egg laying and female survival were not affected after *Rp-dl* pRNAi (Table 2). On the other hand, a severe (98%) decrease in *Rp-dl* mRNA levels led to a 95% reduction in the embryo hatch rate (Table 3). Over 40% of non-hatching embryos resulted from halted development during the early stages, since no germ rudiment was observed, while the majority was comprised of embryos with altered morphologies (Table 4). When we analyzed *Rp-dl* pRNAi embryo morphology, we observed that during blastoderm stages the GR was frequently located at the anterior portion of the egg. Interestingly, diagonal placement of the GR was lost and replaced by perpendicular placement relative to the egg’s long axis (Figure 6A,B). *Rp-Toll* pRNAi lowered the amount of protein in developing oocytes (Additional file 3) and resulted in embryonic phenotypes that resemble *Rp-dl* pRNAi embryos (Table 4).

**Table 2:** **Egg lay resulting from parental RNA interference**

dsRNA (μg/μl)	Egg lay (per female per feeding) ^†^	Number of females
*Rp-dl* (0.5)	31.88 ± 5.35	28
*Rp-cact* (0.5)*	3.5 ± 1.25	10
*Rp-cact* (1.0)*	0.96 ± 0.26	20
*Rp-Toll* (0.5)	38.5	25
Control (0.5)	26.0 ± 1.38	20

^†^Values are average ± standard deviation. *p < 0.01, versus control.

**Table 3:** **Embryo viability resulting from parental RNA interference**

dsRNA (μg/μl)	Embryo viability (%) ^†^	Number of eggs
*Rp-dl* (0.25)	58.51	135
*Rp-dl* (0.5)*	5.5 ± 2.03	801
*Rp-dl* (1.0)*	1.55 ± 0.184	400
*Rp-cact* (0.5)*	3.37 ± 1.71	89
*Rp-cact* (1.0)*	0	123
*Rp-Toll* (0.5)	0.59	168
Control (1.0)	89.54 ± 5.03	514

^†^Values are average ± standard deviation. *p < 0.01, versus control.

**Table 4:** **Embryonic phenotypes resulting from** ***Rp-dl*** **pRNAi**

dsRNA (μg/μl)	Embryonic phenotypes (%)				n
	No germ rudiment*	Embryo anteriorly localized**	Embryo randomly localized	Embryo posteriorly localized	
*Rp-dl* (0.5) stages 2-4	44	50	5	0	18
*Rp-dl* (1.0) stages 2-4	32	52	4	12	25
*Rp-dl* (0.5) stages 5-10	22	68	7	3	59
*Rp-Toll* (0.5) stages 2-10	7	23	30	40	30
control (1.0) stages 2-10	6	-	-	94	50

*“No germ rudiment” phenotype includes empty eggs and halted embryos that do not form a germ rudiment. **At stage 2 embryos anteriorly localized display embryonic rudiment perpendicular to long axis of egg; after stage 5 anteriorly localized embryos are short, with absent or small appendage-like structures (25% penetrant appendage-like phenotype).

**Figure 7:**
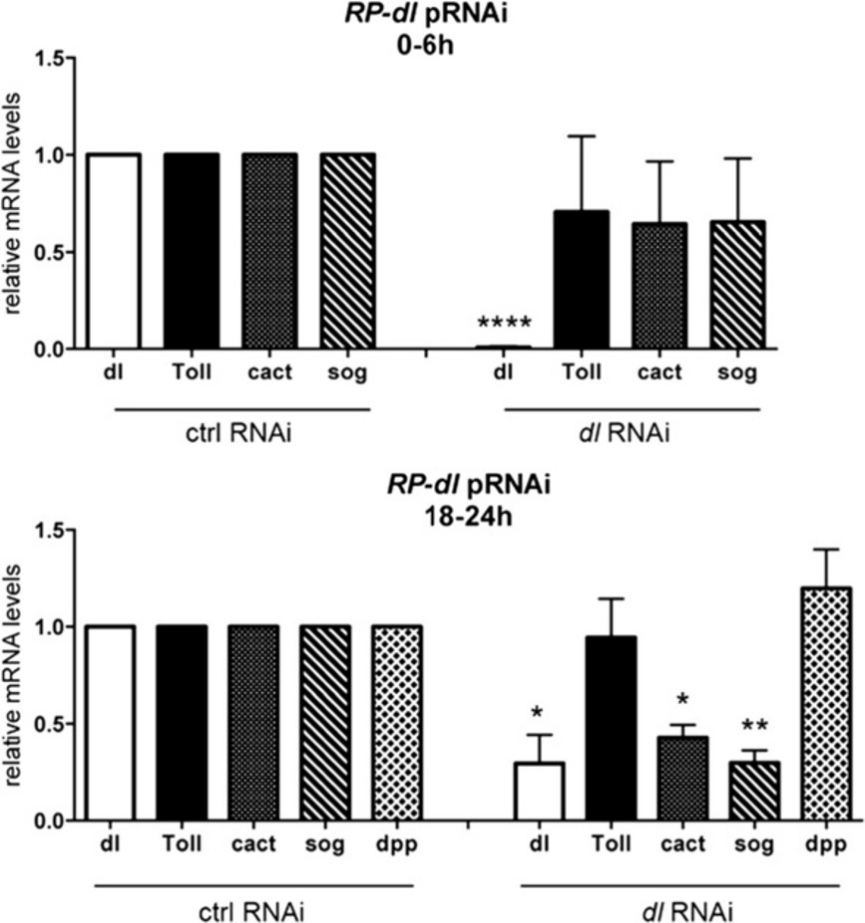
***Rp-sog*** **and** ***Rp-cact*** **mRNA levels are regulated by** ***Rp-dl*** **.** Normalized mRNA levels for *Rp-dl, Rp-Toll, Rp-cact, Rp-sog*, and *Rp-dpp* in control *mal* parental RNA interference (pRNAi) or *Rp-dl* pRNAi (0.5 μg/μL) embryos were evaluated at stage 1 (0 to 6 hours) and stage 2B (18 to 24 hours) embryonic development. *p <0.05, **p <0.01, ****p < 0.0001 by paired *t*-test.

*Rp-dl* pRNAi embryos that reach the segmentation stages are short and mostly mislocalized to the anterior region of the egg (Figure 6C-H). These embryos present a tube-like structure similar to that of *Tc-dl* RNAi embryos, which lack mesodermal cells [5]. Thus, we performed cross-sections of *Rp-dl* pRNAi embryos, and confirmed that they lack an internal (most likely mesodermal) layer (Figure 6I,J). No extraembryonic amnion covering the embryo was observed either. Furthermore, we observed mild *Rp-dl* pRNAi phenotypes in 25% of segmentation stage embryos which were characterized by the presence of vestigial appendages directed towards the surface of the egg (Figure 6E-H). Similar phenotypes have been seen for spider embryos under *sog* pRNAi, which have been suggested to result from the loss of ventral structures [8]. This interpretation is also consistent with the decrease in *Rp-sog* expression we observed during stage 2B caused by *Rp-dl* pRNAi (see below, Figure 7). Likewise, we observed a decrease in *Rp-sna* and *Rp-twi* mRNA levels in *Rp-dl* knockdowns (Figure 6K). Therefore, the phenotypes resulting from loss of Toll signals may be due to loss of ventral embryonic territories.

On the other hand, parental RNAi for *Rp-cact* results in oogenesis phenotypes that preclude embryonic formation: (Figure 5). In order to unveil the role of *cactus* during *Rhodnius* embryogenesis, we performed pRNAi on adult females. Intriguingly, *Rp-cact* dsRNA-injected females showed underdeveloped ovaries and large undigested gut contents 1 week after blood feeding (Figure 5F, data not shown). In contrast, control dsRNA-injected females display ovaries at the choriogenic stage and largely absent gut content (Figure 5E, data not shown). After *Rp-cact* dsRNA, female adult mortality increased with time and reached 100% after 10 days, even when a low dsRNA concentration was adopted for the pRNAi assay (<100 ng/μL). The low number of eggs laid by surviving females (Table 3) precluded the analysis of possible *Rp-cact* during DV formation. Similar adult lethality has been observed in experiments investigating *Tc-cact* in the beetle *Tribolium*[5]. Only the injection of embryonic RNAi, as done in *Tribolium*, would be able to unveil the role of *Rp-cact* during DV axis formation*,* a technique not yet applied in *Rhodnius* due to its hard chorionic structure. This suggests that *Rp-cact* has additional functions that may not involve *Rp-dl* or that excess Toll signals are more deleterious to oogenesis than lack of Toll.

### *Rp-dl*regulates expression of the Bone Morphogenetic Protein pathway gene *Rp-sog*

In order to investigate hierarchical relationships between Toll and BMP pathway genes during embryogenesis, we analyzed the expression of *Rp-Toll*, *Rp*-*cact*, *Rp*-*sog*, and *Rp-dpp* under *Rp-dl* pRNAi. Functional assays for *Rp-dl* showed that a 60 to 80% decrease in *Rp-dl* mRNA levels (Figure 7) results in an approximately 60% decrease in stage 2B *Rp-sog* and *Rp-cact* mRNAs, while stage 1 mRNA levels are unchanged (Figure 7). In contrast, no significant changes in stage 1 mRNA levels were seen for *Rp-dpp, Rp-cact*, and *Rp-Toll* under the same condition (Figure 7). No differences are observed for *Rp-dpp* and *Rp-Toll* at later stage 2B either. Stage 2B mRNAs are likely composed of zygotic messages, since levels are greatly reduced in unfertilized embryos, while stage 1 mRNAs are in great part provided by the mother. Our results strongly suggest that zygotic *Rp-sog* and *Rp-cact* expression during gastrulation (stage 2B) may depend on the activity of the Toll pathway*.*

## Discussion

### *Rp-Toll*is a conserved insect Toll ortholog

In this study, we sought to identify and perform functional analyses of genes required for DV patterning in the hemipteran *Rhodnius*, with the goal of better understanding the evolution of DV patterning in metazoans.

We identified six coding sequences for Toll-related receptors in the *R. prolixus* genome and gut transcriptome (Mesquita and colleagues, manuscript in preparation and [14]), and phylogenetic analysis showed that *Rp-Toll-*2 is the *Toll* ortholog associated with DV patterning during embryogenesis (Figure 1).

In addition to *Toll*, *Drosophila* expresses eight other Toll-related receptor genes that perform redundant or partially redundant functions [40–42]. Phylogenetic analysis of Toll receptors in the mosquito *Anopheles gambiae*, the beetle *Tribolium castaneum*, the bee *Apis mellifera*, the fruit fly *Drosophila melanogaster*, and the bug *Oncopeltus fasciatus* showed major variation among these species [43–45]. In addition, it has been established that *Toll* loci involved in embryonic DV patterning are part of an orthologous group in all insect species studied so far. Therefore, the presence of several *Toll* loci, but only one true Toll ortholog, indicates that the *R. prolixus* genome displays a repertoire of Toll receptors that is characteristic of insects.

In contrast to the receptor diversity, intracellular Toll pathway components appear less diversified in insect evolution. Toll adaptors such as MyD88, Tube, and Pelle display one-to-one orthologs in *R. prolixus* and other insects ( [45]; data not shown). Furthermore, a single IκB Cactus ortholog was identified in the *R. prolixus* genome (this report, [38]). A *dorsal immune factor* (*dif*) ortholog, which codes for a second Rel-like protein in *Drosophila*, is absent in other insects as well as in the *R. prolixus s* genome (M. Sorgine, unpublished results). Thus, Rp-dl and Rp-cact should be effector and inhibitor, respectively, of all Toll functions in *R. prolixus*.

### Toll signals pattern the *Rhodnius*embryonic dorso-ventral axis

Next, we analyzed the effects of parental knockdown on downstream elements of the Toll pathway. Because of the challenges associated with performing embryonic *in situ* hybridization in *Rhodnius*, we conducted an alternative staging protocol, which allowed us to correlate changes in pRNAi gene expression with temporally specific morphological phenotypes. This protocol can be used in future functional analyses conducted during early *Rhodnius* embryogenesis.

*Rp-dl* pRNAi embryos that reach gastrulation stages lack an internal mesodermal layer, which is consistent with a role for Toll signals in DV patterning, as shown for several insect species. Loss of extraembryonic tissues, as shown in our *Rp-dl* knockdowns, has also been associated with defective DV patterning [34, 36, 46–48]. Furthermore, the perpendicular placement of the GR in blastoderm stage *Rp-dl* pRNAi embryos is reminiscent of *Tc-Toll* and *Tc-sog* pRNAi embryos, where it has been suggested that the Toll pathway harbors an AP as well as DV component [5]. Our molecular analysis showed that *Rp-Toll* and *Rp-dl* are expressed during oogenesis and embryonic cleavage stages, and that the mother delivers a great part of the *Rp-Toll* and *Rp-dl* mRNAs for use during embryogenesis. A second peak of *Rp-Toll* (24 to 36 hours) and *Rp-dl* (18 to 30 hours) mRNAs resulting from zygotic expression is observed at gastrulation. Thus, the fundamental question is whether the DV patterning phenotypes observed under *Rp-dl* pRNAi are due to blockage of maternally or zygotically provided *Rp-dl* and *Rp-Toll*.

Maternal messages are fundamental for the development of insect embryos. For example, maternal messages via the Epidermal Growth Factor Receptor pathway break the symmetry in *Drosophila, Nasonia*, and *Tribolium* and *Gryllus bimaculatus* ovarian follicles [49], setting up the embryonic DV axis. In *Drosophila,* this symmetry-breaking event leads to the differential activation of maternally provided Toll receptors along the DV axis, which is translated into the nuclear Dorsal gradient during embryogenesis. Indeed, in this diptera, *Toll*, *dorsal*, and *cact* functions in DV axis formation are exclusively maternal [50].

However, positional information derived from the mother is not a strict requirement. For instance, exposure of *Atrachya* beetle embryos to cold temperatures leads to the production of germ bands that are randomly condensed relative to the AP and DV axes [51], implying that axial patterning is regulated zygotically in this species. Moreover, careful investigation of DV axis establishment in *T. castaneum* showed that Toll and Dl are involved in a zygotic self-regulatory circuit [5]. Evidence for this circuit was provided by the comparison of spatial expression patterns of DV target genes in embryonic versus parental RNAi of *Tc-Toll*, *Tc-dorsal*, and *Tc-cactus*. Embryonic and parental knockdown phenotypes are identical in *T. castaneum*, strongly suggesting that most DV patterning events take place at the zygotic level.

Unfortunately, we were unable to reproduce this level of spatial resolution in the expression of DV genes in *Rhodnius* since we were not able to reproduce the previously published *in situ* protocols [23, 24] and embryonic injections are remarkably difficult to administer due to the hard chorion of *Rhodnius*. However, at the morphological level, early misplacement of GR cells in the anterior of the egg under *Rp-dl* pRNAi suggests that the maternal Toll pathway performs an early axial patterning function that affects later developmental stages. Furthermore, perpendicular placement of the GR relative to the long axis of the egg under *Rp-dl* pRNAi resembles the loss of DV polarity phenotype reported for *Tc-Toll* and *Tc-dpp*[5], suggesting an early effect of *Rp-dl* in DV patterning. During the early stages, the decrease in *Rp-dl* mRNA levels under RNAi is greatest (>90%), with most resulting embryos (84%) being anteriorly misplaced and short. Therefore, it is reasonable to suggest that DV patterning is controlled by maternal *Rp-dl*.

**Figure 8:**
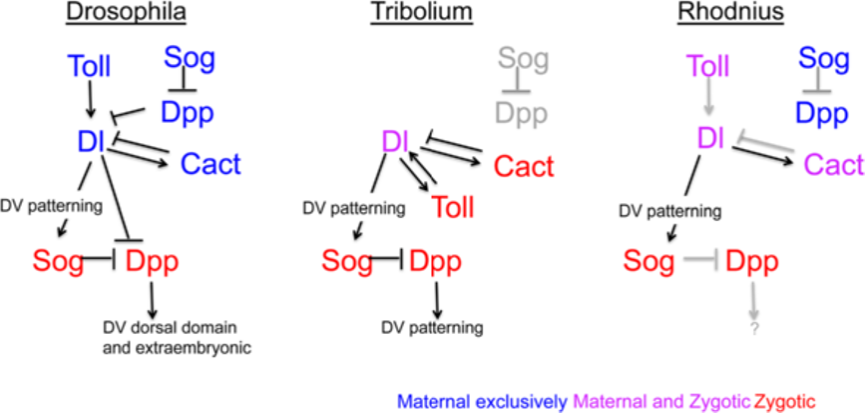
**Model for the conserved action of Toll signals during** ***Rhodnius*** **dorso-ventral patterning.** The hierarchical relationships between Toll and Bone Morphogenetic Protein pathways in dorso-ventral (DV) patterning have been established for *Drosophila* and *Tribolium*, either in terms of gene transcription or protein activity, and are displayed in simplified form to compare with relationships defined here for *Rhodnius*. Neither *Tribolium* nor *Rhodnius* Toll pathways regulate *dpp* expression, activity required to pattern the dorsal region of the *Drosophila* embryo. On the other hand, a feedback loop between Toll and dl is only present in *Tribolium*. In the three species analyzed, *dl* positively regulates *sog* expression. Arrows in black are those tested experimentally. Arrows in grey denote relations that are assumed based on conserved aspects and published evidence throughout the animal kingdom. Maternal, zygotic or maternal + zygotic expression are displayed in blue, red and pink, respectively. Maternal (blue) and zygotic (red) expression of *dpp* and *sog* are not gathered as one in *Drosophila* and *Rhodnius* since their epistatic relationships to the Toll pathway differ (*Drosophila*), or have not been fully analyzed (*Rhodnius*).

In conclusion, while zygotic *Tc-Toll* seems to be the central player in *Tribolium* DV patterning, and the role for *Drosophila Toll* in embryonic patterning is exclusively maternal, maternal *dl* in both insects regulates establishment of the DV axis. Our results support the notion of a conserved DV patterning function for maternal *Rp-dl*. Furthermore, the major differences between DV patterning among these three species seem to reside in the maternal versus zygotic contribution of Toll signals, and the presence or absence of positive feedback loops (Figure 8).

### *Rhodnius*Toll signals play a novel role in defining the embryo’s position along the anterior-posterior axis

Interestingly, our functional assays reveal that *Rp-dl* pRNAi leads to a severe defect in the positioning of the developing embryo, which is found at the anterior of the egg instead of being placed at the posterior. This observation strongly suggests that the Toll pathway exerts a role not only in the establishment of the DV axis, but also in the AP axis. The embryo’s anterior placement could result from incorrect specification of extraembryonic membranes, since we observed a small number of amniotic cells in *Rp-dl* RNAi embryos. However, we observed misplaced embryos as early as the syncytial blastoderm stages, before extracellular membranes are able to exert a tracking effect on the developing embryo. Therefore, an alternative hypothesis is that specification of the AP and DV axes are interdependent and that *Rp-dl* RNAi interferes with the expression of AP patterning genes. A recent study reported that patterning of the two major body axes is co-dependent in *Apis mellifera*. Considering that we found *Rp-dl* pRNAi embryos mislocalized along the AP axis, a similar mechanism may play a role during axis specification in *Rhodnius*[10].

Anteriorly placed *Rhodnius* embryos are short and do not emerge into the yolk, suggesting that anatrepsis has been disturbed. Such an effect has not been previously described for the Toll pathway in any of the arthropod species analyzed to date. However, it has been recently reported that interfering with the terminal system in the hemipteran *Oncopeltus* disturbs invagination of the blastoderm although, in this case, germ band extension and segmentation proceeds almost normally [52]. This is consistent with the interpretation that part of the phenotypes displayed by interfering with the Toll pathway in *Rhodnius* may result from AP patterning defects.

### Respective contribution of the Toll and Bone Morphogenetic Protein pathways to *Rhodnius*dorso-ventral patterning

A long-standing question in embryonic patterning concerns identifying the point in evolution at which the two main pathways (Toll and BMP) were deployed to pattern the DV axis, as well as elucidating their respective contributions along different clades. Studies in ancestral arthropod classes (for example, the crustacean *Parhyale hawaiensis*[53] and the spider *Parasteatoda tepidariorum*[8]) indicate no role of Toll in DV patterning, although Toll is detected during embryogenesis of the tick *Riphicephalus (Boophilus) microplus*[54]. In fact, in these arthropods, BMPs seem to perform a more central role in setting the DV axis.

The role of Toll in immunity has been functionally assayed in several insects (see references in [55]), unlike its role in DV patterning, which has only been studied in a few holometabolous insects [2, 10, 56] and more recently in *Oncopeltus* (J.A. Lynch and S. Roth, personal communication). The functional analyses of *Rp-dl* conducted in this study show that the Toll pathway plays a role in *Rhodnius* embryogenesis. However, *Rp-dl* and *Rp-cact* are also expressed in the salivary gland, intestine, and fat body, where they play a role in the response to infection ( [38], this report). Therefore, considering the hypothesis that the ancestral function of the Toll pathway is in immunity [2], our results support the notion that acquisition of an embryonic role for Toll predates the common ancestor of hemiptera and hymenoptera.

The relative contribution of the Toll and BMP pathways to DV patterning varies across species. In vertebrates, BMPs perform a central role in setting the DV axis, and no functional role for an NFκB pathway in this context has been reported [57]. In insects, sequences that respond to Dorsal/NFκB have been identified, which drive DV restricted expression patterns in *Anopheles*, *Drosophila*, and *Tribolium*[46, 58–61]. In *Drosophila,* maternal Toll signals play the greatest role in defining territories along the DV axis, spatially restricting the zygotic expression of target genes such as *dpp* and *sog*. Sog and Dpp proteins subsequently subdivide the dorsal domain and drive the formation of the extraembryonic amnioserosa [48, 62]. At the beginning of gastrulation in the scuttle fly *Megascelia*, BMP subdivides the extraembryonic amnion and serosa as well [47]. In contrast, in *Tribolium*, both the Toll and BMP pathways set the entire DV axis: loss of Tc-Dpp signals causes loss of extraembryonic (dorsal-anterior) tissue and embryo expansion, while a decrease in *Tc-Toll* or *Tc-sog* results in expansion of dorsal-anterior structures at the expense of embryonic tissue [5, 63, 64].

In *Rhodnius*, the presence of maternal *Rp-Toll* and *Rp-dl* mRNAs in cleavage stage embryos, the absence of *Rp-dpp* expression at this stage, and the decrease in zygotic *Rp-sog* in *Rp-dl* pRNAi, suggest that maternal Toll sets the initial events in embryonic DV patterning, as shown for *Drosophila* (Figure 8). On the other hand, the presence of maternal *Rp-sog* messages during cleavage stages suggests an early function for *Rp-sog* that may differ from Sog function in either *Drosophila* or *Tribolium*. While no *Rp-dpp* expression is observed at these early stages, Rp-Sog may exert an effect on other BMPs such as Rp-Gbb, which is yet to be explored.

The lack of a central role for *Rp-dpp* in setting the DV axis in *Rhodnius* could argue against the hypothesis that the ancestral pathway responsible for DV pattering relied on BMPs. However, we have previously shown that in *Drosophila*, where maternal Toll signals initiate patterned gene expression along the DV axis, maternal expression of *dpp*[65, 66] and *sog*[65] in follicle cells impacts DV axis formation, thus modifying the Dl gradient in the *Drosophila* early embryo [65, 67]. Therefore, despite the fact that *Drosophila* embryos rely on Toll and Dl activation as the main DV patterning event, there might still be a hidden component of maternal *dpp* in DV patterning that corresponds to an ancestral BMP function. Interestingly, we observed that *Rp-sog* and *Rp-dpp* are highly expressed during oogenesis (not shown). Thus, they may perform a patterning function in the follicular epithelium during oogenesis, or provide maternal messages and/or protein that may reflect on embryonic patterning as well. Future functional assays for BMP pathway genes should help clarify the role of the BMP pathway during *Rhodnius* embryogenesis, and provide insights as to the relative contribution of the BMP and Toll pathways in this hemiptera.

## Conclusions

In this study, we investigated the role of the Toll pathway in *Rhodnius prolixus* embryonic development. We show that the Toll pathway presents maternal and zygotic components. Functional assays revealed that *Rp-dl* determines early placement of the germ rudiment at the egg’s posterior. In addition, embryos resulting from *Rp-dl* pRNAi lack a mesodermal layer and display decreased amnion. Thus *Rp-dl* is necessary for DV patterning. While *Rp-dpp* mRNAs are absent in early embryonic stages, we observe that the Toll pathway regulates zygotic expression of *Rp-sog* preceding gastrulation. In conclusion, the Toll pathway is a central component in *Rhodnius* embryonic DV patterning.

## Electronic supplementary material

Additional file 1: **Primer list and quantitative PCR constitutive gene analysis.** (PDF 166 KB)

Additional file 2: **Protein sequence alignment for Rp-Toll paralogs.** (PDF 1 MB)

Additional file 3: **Anti-Toll d300 specifically recognizes** ***R. prolixus*** **Toll protein.** (PNG 2 MB)

Additional file 4: ***Rp-dpp*** **expression during early embryogenesis.** (PDF 263 KB)

## Abbreviations

AP: anterior-posterior
DV: dorso-ventral
BMP: Bone Morphogenetic Protein
bp: base pair
sog: *short gastrulation*
*chd*: *chordin*
*dl*: *dorsal*
*dpp*: *decapentaplegic*
GR: germ rudiment
NF: nuclear factor
PBS: phosphate-buffered saline
PCR: polymerase chain reaction
PFA: paraformaldehyde
pRNAi: parental RNA interference
Rp: *Rhodnius prolixus*
Tc: *Tribolium castaneum*.
