# Supplementary material for: Toll signals regulate dorsal–ventral patterning and anterior–posterior placement of the embryo in the hemipteran Rhodnius prolixus
Source: EvoDevo. 2014 Oct 27;5:38. doi: 10.1186/2041-9139-5-38 (PMC4407881; doi:10.1186/2041-9139-5-38)
Supplement: Supplementary file 1 — Additional file 1: Primer list and quantitative PCR constitutive gene analysis. (PDF 166 KB) [file 13227_2014_133_MOESM1_ESM.pdf]

Additional Tables

Additional Table 1. Ranking of candidate reference gene tested by BestKeeper, geNorm and NormFinder. The best gene for housekeeping during *Rhodnius* embryonic stages was chosen by ranking analysis using the BestKeeper, geNorm and NormFinder programs. For the analysis of best candidate genes with BestKeeper, we ranked the genes according to the coefficient of correlation (r) and the p-value between each candidate gene and the BestKeeper index (Pfaffl et al., 2004). The geNorm program estimates gene expression stability (M value). The candidate gene with the lowest M value is considered to have the most stable expression (Vandesompele et al., 2002). In addition, according to NormFinder, the candidate genes that are more stably expressed are indicated by lower expression stability values (Andersen et al., 2004). Considering the whole dataset, *Rp-18S* and *Rp-Ef1* were the most stable genes.

Gene ranking	BestKeeper		geNorm	NormFinder
	coef. corr. (r)	*p-value*	stability value (M)	stability value
Rp-18S	0.965	0.001	0.779	0.136
Rp-Ef1	0.930	0.001	0.725	0.172
Rp-gadph	0.773	0.024	0.856	0.495
Rp-mip	0.247	0.558	1.224	0.749

Additional Table 2. qPCR primer sequences used in this study.

Rp-Gene	Primers (5'-3')		Amplicon size (bp)
	**Forward**	**Reverse**	
dl	TGTCGGTTTAGCAGCTGTTGGT	TCCAGATGGCTTTGAAAAATCCCTG	95
Toll	TCTTGACCCGGAGCTGAAAGC	CCGTAGGATGTGGCAAGGCA	104
cact	GTGCTGGTGCTTGTACGAAA	GGAGTCGGACGATACCTCAA	154
dpp	ACAGCGCAAAAAGGATGGACG	TCGGCCAATGGAAATGGGCA	147
sog	TGTTTGCCACAGCCAACCGA	GTCAGCCAAGGAACGCCACT	141
18S	TCGGCCAACAAAAGTACACA	TGTCGGTGTAACTGGCATGT	104
Ef1	GATTCCACTGAACCGCCTTA	GCCGGGTTATATCCGATTTT	92
gadph	TTTTGATGCAAAAGCTGGAA	AACGACACGATTTGAATAGCC	94
mip	CCTTCTTGGTGGACCCTACA	TCCAACCCAGTACACCCAAT	106
sna	AAACAACATCCTCGTCCGCT	TGTTGATGTTTTGACAGTCCGG	100
twi	GGCTAATGTAAGAGAGCGCC	GATGCCAACCTCAGTGTTTGT	123

Rp-gadph (*gliceraldehyde-3-phosphate dehydrogenase*) and *Rp-mip* (*major intrinsic protein*) primers were a gift from Dr. D. Majerowicz. All other genes are referred to in the main text.

Additional Table 3. pRNAi primer sequences used in this study. Nucleotides in small caps correspond to the T7 sequences.

Rp-Gene	Primers (5'-3')		Amplicon size (bp)
	**Forward**	**Reverse**	
dl	ggccgcggAAGAGGACATATCAGTGCGATTC	cccggggcTGTTGAAAACGTACGGTAGTCG	303
Toll	ggccgcggTCTTGACCCGGAGCTGAAAGC	cccggggcCCGTAGGATGTGGCAAGGCA	718
cact	ggccgcggTGCTTACTCTTTAAGGCAGTCC	cccggggcGGAGTCGGACGATACCTCAA	755

References:

Andersen CL, Jensen JL, Orntoft TF: **Normalization of real-time quantitative reverse transcription-PCR data: a model-based variance estimation approach to identify genes suited for normalization, applied to bladder and colon cancer data sets**. Cancer Res 2004, 64:5245-5250.

Pfaffl MW, Tichopad A, Prgomet C, Neuvians TP: **Determination of stable housekeeping genes, differentially regulated target genes and sample integrity: BestKeeper-Excel-based tool using pair-wise correlations.** Biotechnol Lett 2004, 26:509-515.

Vandesompele J, De Preter K, Pattyn F, Poppe B, Van Roy N, De Paepe A, Speleman F: **Accurate normalization of real-time quantitative RT-PCR data by geometric averaging of multiple internal control genes.** Genome Biol 2002, 3:1-11.
