# Supplementary material for: Toll signals regulate dorsal–ventral patterning and anterior–posterior placement of the embryo in the hemipteran Rhodnius prolixus
Source: EvoDevo. 2014 Oct 27;5:38. doi: 10.1186/2041-9139-5-38 (PMC4407881; doi:10.1186/2041-9139-5-38)
Supplement: Supplementary file 2 — Additional file 2: Protein sequence alignment for Rp-Toll paralogs. (PDF 1 MB) [file 13227_2014_133_MOESM2_ESM.pdf]

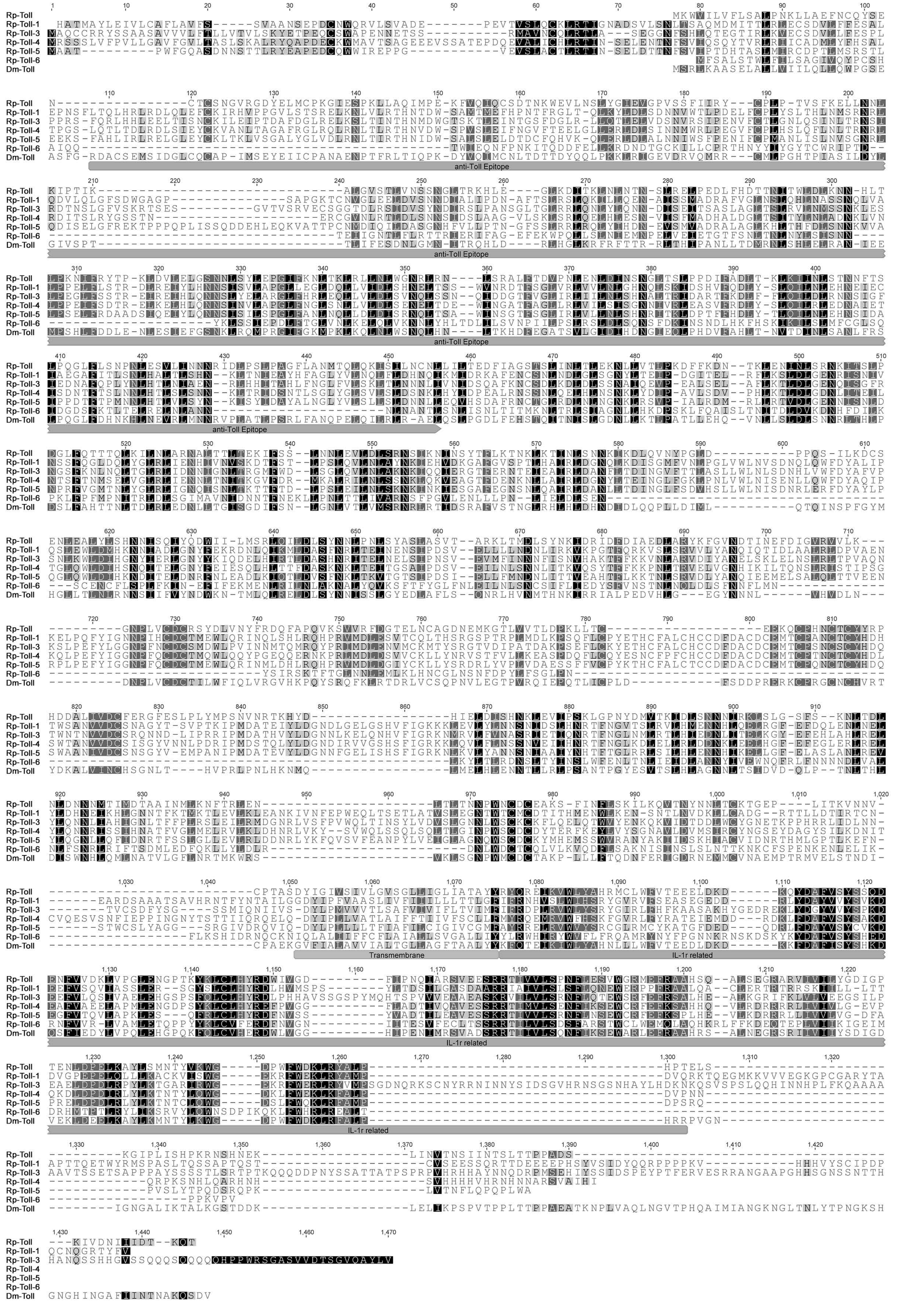

Additional Figure 2. Protein Sequence alignment for Rp-Toll paralogs. Predicted protein sequences for Rp-Toll-2 (Rp-Toll) are aligned with *R. prolixus* Toll paralogs. Light grey to dark grey shading denotes increase in sequence similarity. Region underlined in grey corresponds to the epitope recognized by the anti-Toll antiserum d300 (aa 108-455), and to IL-1-related (aa 1051-1302) region.
