# Supplementary figures and images for: Toll signals regulate dorsal–ventral patterning and anterior–posterior placement of the embryo in the hemipteran Rhodnius prolixus
Source: EvoDevo. 2014 Oct 27;5:38. doi: 10.1186/2041-9139-5-38 (PMC4407881; doi:10.1186/2041-9139-5-38)

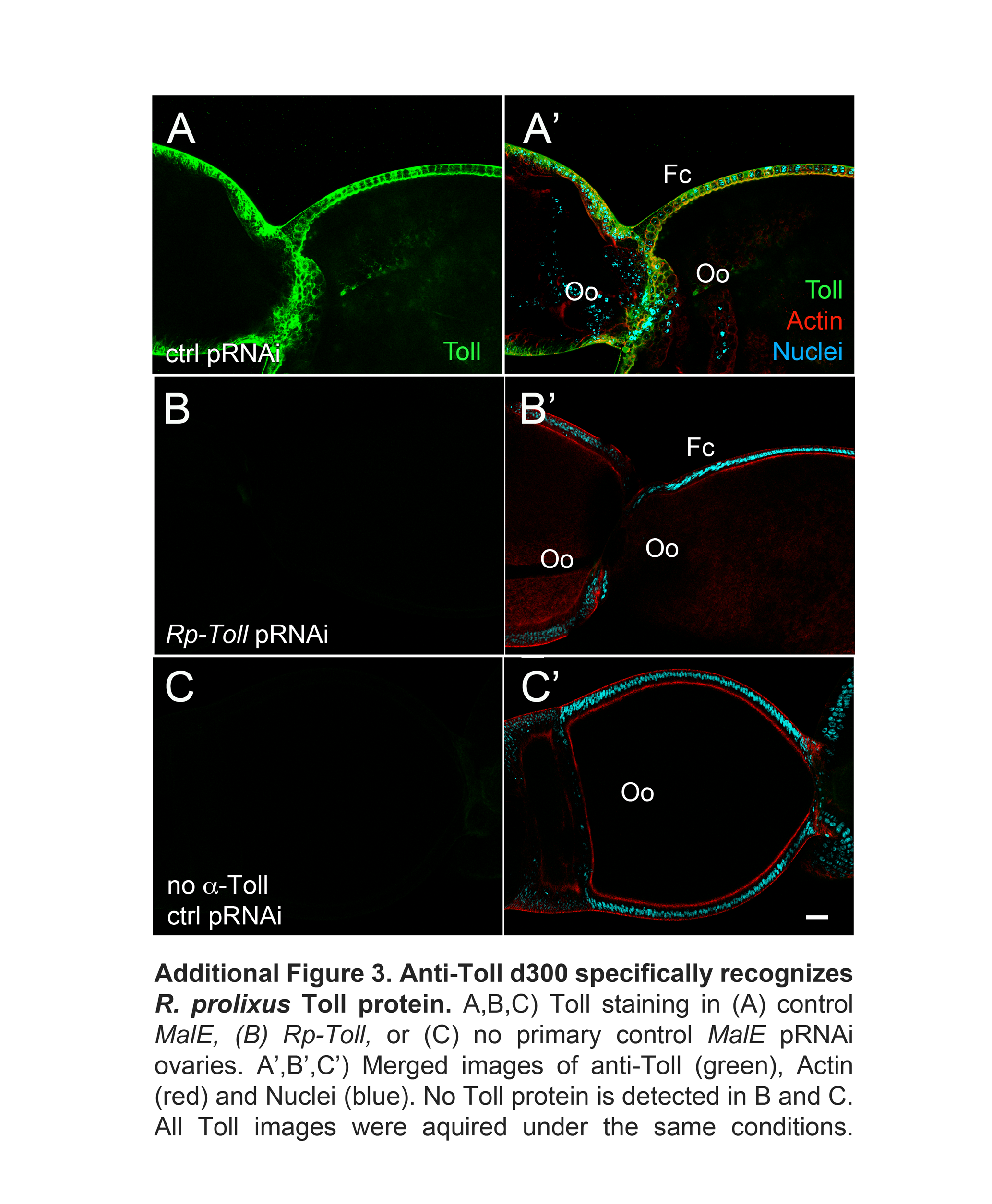

Supplement: Supplementary file 3 — Additional file 3: Anti-Toll d300 specifically recognizes R. prolixus Toll protein. (PNG 2 MB) [file 13227_2014_133_MOESM3_ESM.png]
