# Supplementary material for: Toll signals regulate dorsal–ventral patterning and anterior–posterior placement of the embryo in the hemipteran Rhodnius prolixus
Source: EvoDevo. 2014 Oct 27;5:38. doi: 10.1186/2041-9139-5-38 (PMC4407881; doi:10.1186/2041-9139-5-38)
Supplement: Supplementary file 4 — Additional file 4: Rp-dpp expression during early embryogenesis. (PDF 263 KB) [file 13227_2014_133_MOESM4_ESM.pdf]

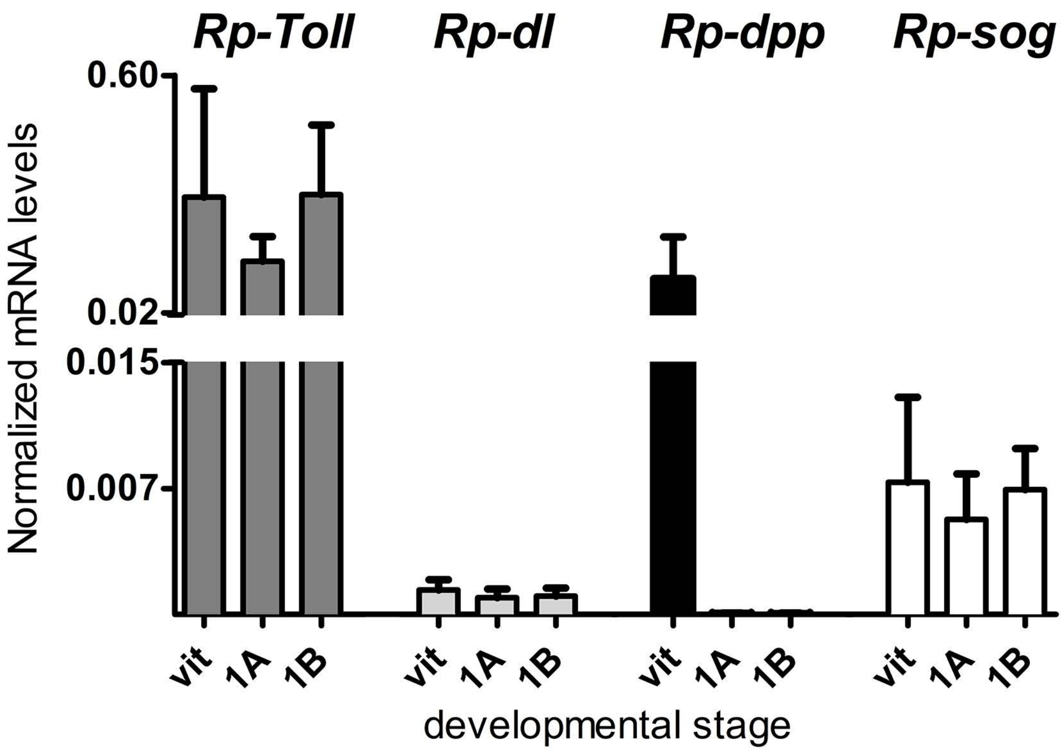

Additional Figure 4. Normalized mRNA levels for *Rp-Toll*, *Rp-dl*, *Rp-dpp* and *Rp-sog* during vitelogenesis (vit) and early embryonic stages (1A and 1B). Absolute mRNA levels for each gene were normalized to *Ef1* and plotted in logarithmic scale to show differences in levels between the different genes. This analysis show that *Rp-dpp* is expressed during oogenesis but the mRNA is not transferred to the embryo. No *Rp-dpp* expression is seen at the early stages that could result in effects along the embryonic DV axis.
